# Establishing a Multicenter Active Adverse Events Following Immunization Sentinel Surveillance Network Across 22 Tertiary Care Hospitals in India: Protocol for a Prospective Observational Study

**DOI:** 10.2196/64050

**Published:** 2025-08-08

**Authors:** Apoorva Sharan, Manoja Kumar Das, Akhil PM, Ramesh Poluru, Neeraj Kumar Kashyap, Christian Burri, Jan Bonhoeffer, Satinder Aneja, Deepak Polpakara, Narendra Kumar Arora, MAASS Study Group

**Affiliations:** 1 University of Basel, Basel Switzerland Swiss Tropical and Public Health Institute (Swiss TPH), Allschwil, Switzerland Inclen Trust International New Delhi India; 2 Inclen Trust International New Delhi India; 3 Swiss Tropical and Public Health Institute University of Basel, Basel Switzerland Allschwil Switzerland; 4 University of Basel Children's Hospital University of Basel, Basel, Switzerland Basel Switzerland; 5 School of Medical Sciences & Research, Sharda University Greater Noida India; 6 John Snow India Private Limited New Delhi India

**Keywords:** vaccine safety, pharmacovigilance, multicenter studies, adverse events following immunization, low- and middle-income countries, postlicensure safety surveillance, active surveillance

## Abstract

**Background:**

The rapid evolution of immunization programs in low- and middle-income countries (LMICs) has necessitated an augmentation of capacity for postlicensure vaccine safety monitoring.

**Objective:**

This study describes the protocol for establishing a Multicenter Active Adverse Events Following Immunization Surveillance System (MAASS) network in India, which conducted prospective observational surveillance for 12 adverse pediatric outcomes between November 1, 2017, and March 20, 2020.

**Methods:**

A multistage site selection process was implemented, beginning with an initial screening survey followed by in-person visits to assess the suitability of potential tertiary care hospitals for inclusion in the network. We adopted a decentralized, collaborative approach to develop the study protocol, standardize case definitions, establish data collection procedures, and create a common data model for monitoring and analysis. Outcomes selected for surveillance included acute disseminated encephalomyelitis, anaphylaxis, aseptic meningitis, dengue, Guillain-Barré syndrome, Kawasaki disease, malaria, seizure, sepsis, thrombocytopenia, intussusception, and urinary tract infections. We screened all children aged 1-24 months who were hospitalized for more than 24 hours at participating sites to identify suspected or confirmed cases of these outcomes using a structured checklist. Written informed consent was obtained from the parent or legally authorized representative for inclusion in the study. Demographic, socioeconomic, and vaccine exposure information was collected for all included participants. Additional clinical information was gathered to assess the level of diagnostic certainty according to standardized case definitions. The study progressed through 3 distinct phases: network establishment (January-November 2017), active surveillance (November 2017-March 2020), and database analysis (April 2020-March 2024). The dissemination process is currently underway.

**Results:**

A geographically representative data network was established across 15 public and 7 private tertiary care hospitals in 17 states and 1 union territory in India. During the study period, we screened 90,147 age-eligible admissions and confirmed 8362 cases with study outcomes. Using multiple analytic study designs, we generated a database of outcomes and exposures to investigate associations between vaccine-event pairs of interest.

**Conclusions:**

The MAASS network is unprecedented in its scope and scale among LMICs. While the study is specific to India, the lessons learned in establishing and implementing the network offer valuable insights for developing active surveillance systems and strengthening capacity for benefit-risk evaluations of vaccines in resource-constrained settings.

**International Registered Report Identifier (IRRID):**

RR1-10.2196/64050

## Introduction

### Background

The COVID-19 pandemic highlighted significant health system inequities, including in vaccine safety surveillance. Nearly two-thirds of all active vaccine safety surveillance evaluations were conducted in high-income countries, underscoring the limited capacity for postauthorization vaccine safety monitoring in low- and middle-income countries (LMICs) [1]. To help avert future pandemics, the Coalition for Epidemic Preparedness Innovations’ 2022-2026 strategy aims to develop safe and effective vaccines against potential diseases within 100 days [2]. Robust vaccine pharmacovigilance systems are critical for ensuring effective vaccine monitoring, particularly because, even under routine conditions, clinical trials lack the power to detect rare and long-term adverse events following immunization (AEFIs) [3]. Clinical trials do not reflect real-world immunization conditions, where vaccines are administered to diverse populations. Without strong postlicensure surveillance, safety concerns could undermine public trust and disrupt immunization programs [4,5].

As more vaccines are tailored to the needs of LMICs, progress in strengthening vaccine pharmacovigilance capacity has not kept pace with the rapid evolution of immunization programs, despite systematic efforts [6,7]. Most LMICs rely on passive reporting systems that often miss or underreport adverse events [8,9]. These systems also lack comparable data from unvaccinated cohorts. The limited availability of clinical information hampers case verification, making it difficult to confirm potential vaccine-event links [10]. To address these gaps, the World Health Organization’s (WHO) Global Vaccine Safety Blueprint documents (versions 1 and 2) call for strengthening capacity for active and hospital-based sentinel safety surveillance at the national, regional, and global levels [11,12]. Active vaccine safety surveillance systems proactively solicit reports of events, enabling the systematic investigation of very rare (ie, fewer than 1 event per 10,000 doses) or long-term sequelae after vaccination [13]. Unlike passive surveillance, which depends on spontaneous reporting, active surveillance uses standardized case definitions and protocols to systematically identify adverse events, allowing for more complete detection of rare or underreported events. In LMICs, where health care infrastructure and routine reporting capacity may be limited, active surveillance helps bridge critical gaps in passive systems, thereby supporting more comprehensive vaccine safety monitoring [12,14]. In 2016, the Ministry of Health and Family Welfare tasked the International Clinical Epidemiology Network (INCLEN) with establishing the Multicenter Active AEFI Surveillance System (MAASS) to complement ongoing efforts to improve the coverage, quality, and safety of the national immunization program. This paper describes the protocol for establishing a prospective observational surveillance network to monitor adverse pediatric outcomes in resource-constrained settings.

### Study Objectives

This study aims to establish a sustainable, geographically and socioculturally representative hospital-based sentinel surveillance network capable of identifying and classifying pediatric outcomes and documenting vaccine exposure status to inform benefit-risk evaluations of routinely administered vaccines in India. The specific objectives were to (1) estimate baseline hospital outcome rates (per 100,000 pediatric admissions between 1 and 24 months of age per year) for the study outcomes; (2) characterize the seasonality, as well as the demographic, socioeconomic, and clinical profiles, and the vaccine exposure status of selected study outcomes; (3) assess the applicability of standardized case definitions (eg, Brighton Collaboration definitions for known AEFIs) for study outcomes, using information documented during routine clinical practice and patient care at network sites; and (4) investigate the association between selected vaccine-event pairs using appropriate analytical study designs, including case-control, self-controlled case series, and self-controlled risk interval designs.

## Methods

### Design

We developed an operational framework to establish an active surveillance network for monitoring AEFIs in tertiary care hospitals across India. The framework aims to estimate baseline hospitalization rates for pediatric conditions and document vaccine exposure in children aged 1-24 months.

### Network Governance and Decision-Making

We adopted a decentralized, collaborative approach to governance and decision-making. This included the cocreation of the study design, harmonized data collection, shared responsibility for data quality and management, development of a common data model, and joint formulation of the statistical analysis plan and data interpretation. The study was coordinated and monitored by the Executive Office of the INCLEN Trust International in New Delhi, India. The team at INCLEN developed the zero drafts of the study documents (eg, protocol, operating procedures, tools, statistical analysis, and data management plans), organized protocol finalization workshops, conducted training, monitored data quality, and analyzed and interpreted the results. Dedicated research staff (n=2) collected data at each surveillance site, while faculty from the departments of pediatrics, pediatric surgery, and community medicine served as principal and coprincipal investigators. They facilitated all aspects of study implementation and monitored progress throughout the study. The sites served as data custodians; however, in accordance with national guidelines, data ownership remained with the patients [15].

A technical advisory group (TAG)—comprising representatives from the Immunization Division, Ministry of Health and Family Welfare, and the national AEFI committee (n=2), along with an interdisciplinary team of pediatricians (n=4), epidemiologists (n=4), statisticians (n=3), and pharmacovigilance experts (n=3)—provided technical guidance throughout all stages of project implementation.

### Network Establishment

#### Site Selection

**Figure 1:**
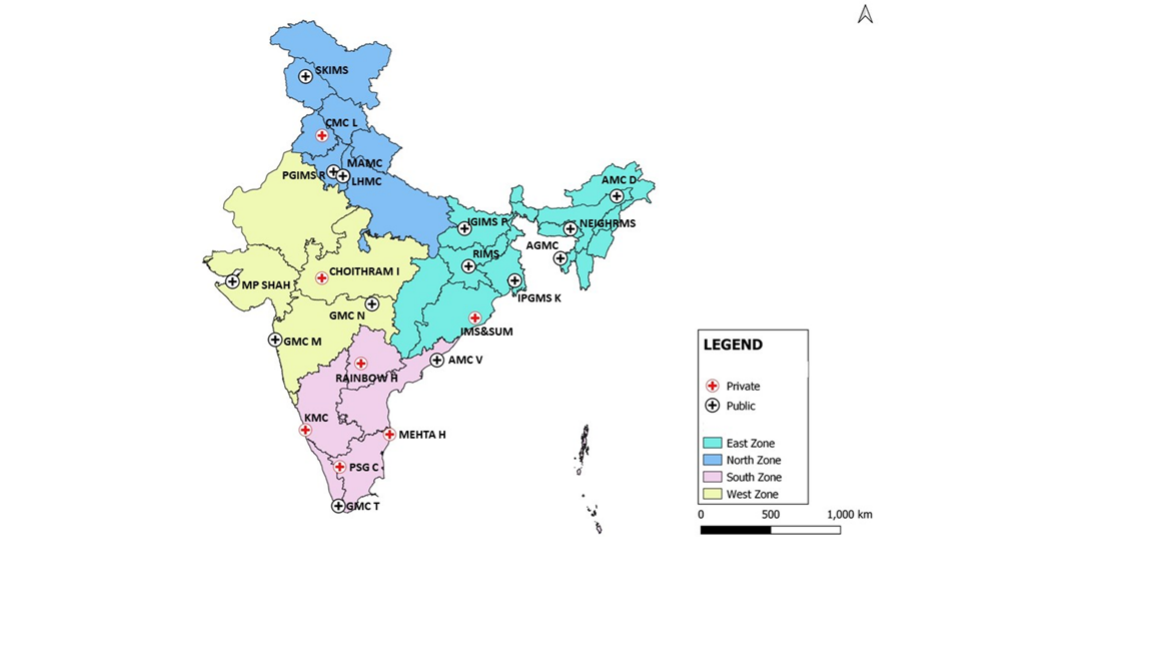
Types of facility in the Multicenter Active AEFI Surveillance System (MAASS) network and their geographic distribution. AGMC: Agartala Government Medical College; AMC D: Assam Medical College, Dibrugarh; AMC V: Andhra Medical College, Visakhapatnam; CMC L: Christian Medical College, Ludhiana; GMC M: Grant Govt. Medical College & Sir J. J. Group of Hospitals, Mumbai; GMC N: Government Medical College Nagpur; GMC T: Government Medical College, Thiruvananthapuram; H: Hospital; IGIMS P: Indira Gandhi Institute of Medical Sciences; IMS & SUM: Institute of Medical Sciences & Sum Hospital; IPGMS K: Institute Post-Graduate Medical Education and Research and Seth Sukhlal Karnani Memorial Hospital, Kolkata; KMC: Kasturba Medical College; LHMC: Lady Hardinge Medical College; MAMC: Maulana Azad Medical College; MP SHAH: Shri M. P. Shah Medical College; NEIGHRMS: North Eastern Indira Gandhi Regional Institute of Health and Medical Sciences; PGIMS R: Pandit Bhagwat Dayal Sharma Post Graduate Institute of Medical Sciences, Rohtak; PSG C: PSG Institute of Medical Sciences and Research, Coimbatore; RIMS: Rajendra Institute of Medical Sciences; SKIMS: Sher-e-Kashmir Institute of Medical Sciences.

**Figure 2:**
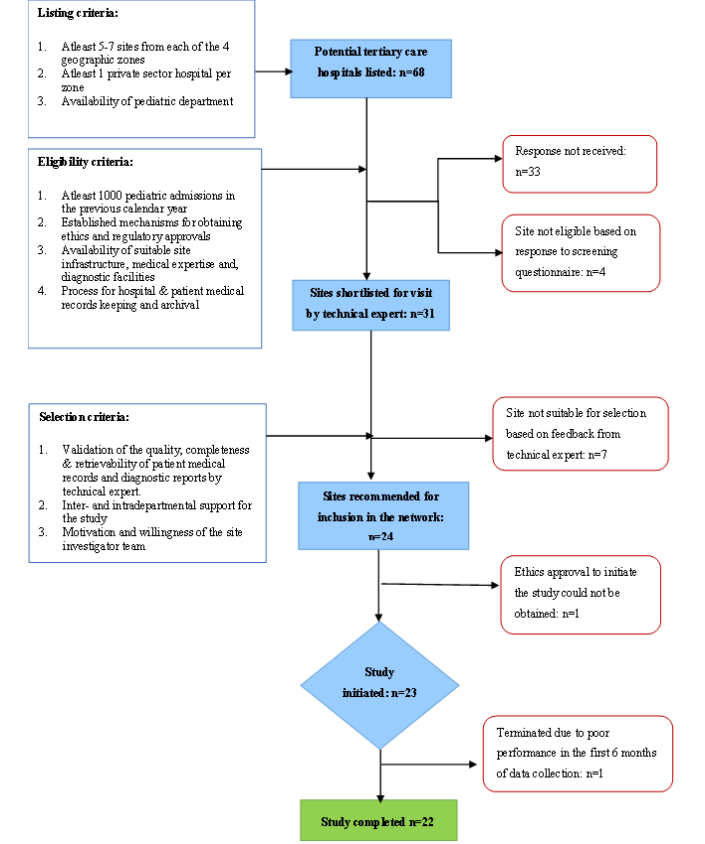
Site selection criteria.

We identified potential sites for inclusion in the network through a systematic, multistage process. INCLEN has been conducting large multicentric studies since 1997 and has fostered partnerships with many medical schools and tertiary care hospitals across the country [16-19]. First, a list of 68 large public and private tertiary care hospitals was developed, based on criteria such as geographic and sociocultural representativeness, the availability of pediatric departments, and, where applicable, the performance of hospitals in prior prospective surveillance studies [19,20]. All listed sites were invited to participate in a screening survey to assess eligibility for inclusion based on predefined criteria (refer to Multimedia Appendix 1 for site selection questionnaires). The survey also helped delineate the ethics and regulatory approval processes, data-sharing policies, and timelines at each potential site. Based on the responses, technical experts shortlisted 31 sites for in-person visits. These visits served to validate findings from the screening survey; assess the quality, completeness, and ease of retrieval of patient medical records and diagnostic reports; and gather qualitative feedback regarding institutional support and willingness to participate in the study. The TAG reviewed the findings from the screening questionnaire and site visits during a review meeting in March 2017 and recommended the selection of 24 sites for inclusion in the network. The sites were chosen to ensure that nearly equal numbers of hospitals were represented from each of the 4 geographic zones of the country (north, south, east, and west), with at least one hospital from the private sector, as illustrated in Figure 1. Figure 2 outlines the eligibility criteria and site selection process followed in establishing the network.

#### Protocol Development

The central team, TAG, and site investigators codeveloped the study protocol during a 3-day workshop held in New Delhi, India (May 2017). Participants provided feedback on the selection of study outcomes, the standardization of case definitions for assessing the level of diagnostic certainty, data sources, and collection procedures, which informed subsequent revisions to the study protocol. Drawing on clinical experience, site investigators developed dummy patient records to pilot-test the draft study tools. The workshop also helped clarify the anticipated technological and human resource requirements, as well as mechanisms for data quality assurance for the study.

#### Training and Study Initiation

We developed a standardized training agenda and supporting documents to promote a uniform understanding of the study protocol, data collection tools, use of the electronic data collection tool, and related procedures. In October 2017, 3 regional training workshops were conducted at network sites in the north (Lady Hardinge Medical College, New Delhi), east (Institute of Post-Graduate Medical Education and Research and Seth Sukhlal Karnani Memorial Hospital, Kolkata), and south (Government Medical College, Thiruvananthapuram) zones to facilitate hands-on training in data collection. Special role-play sessions helped clarify proper procedures for obtaining informed consent and conducting patient interviews. Each site also listed potential source documents and mapped the flow of data collection within their respective settings in preparation for study initiation.

Sites began data collection once training was complete and the necessary ethics and administrative approvals were in place; 17 of the 23 participating sites initiated data collection in November 2017, followed by 3 more sites in December. The final 3 sites began data collection in January, February, and May 2018, respectively.

#### Ethical Considerations

Independent ethics committees at INCLEN (2017/IIEC-052) and 23 of the 24 participating sites approved the master study protocol following the May 2017 workshop. Unanticipated delays in obtaining ethics approval from the twenty-fourth site prevented its participation. In accordance with state norms, additional administrative approvals were obtained from the relevant institutional, state, and national departments before initiating data collection.

The study was conducted in accordance with the principles of the Declaration of Helsinki and the National Ethical Guidelines for Biomedical and Health Research Involving Human Participants [21]. Parents or legally authorized representatives of all potential participants were informed about the voluntary nature of participation, and written informed consent was required for recruitment. The study information sheet and informed consent forms clarified that participation would not result in any direct benefit or harm, nor would it alter the routine medical care of patients. Personal identification and contact information were collected for enrolled participants and retained at the respective sites to enable follow-up of patient records across multiple hospital departments. Source documents validating immunization exposure history were also collected. To maintain confidentiality, robust security measures were implemented during data collection, analysis, and archiving. Paper case report forms (CRFs), source documents, and signed copies of study information sheets and informed consent forms were stored in secure locations at both the study sites and the central data center, with access restricted to core investigators. Electronic CRFs (e-CRFs) were stored in an encrypted format on a secure cloud server with backup and safety features. Personal identification information was removed from the database before analysis, and study participants were identified solely by study-generated unique identification numbers. In accordance with national guidelines [21], study data are securely archived for 5 years.

### Study Procedures

#### Study Population

The study population included all children aged 1-24 months who were hospitalized for any reason and for more than 24 hours in the pediatrics and pediatric surgery departments at network sites. This population was screened for the outcome conditions and also served as the denominator for calculating rates. Most vaccines under the Universal Immunization Programme are administered during this period. The neonatal period was excluded, as only a few vaccines are administered during this time, and sampling and diagnostic challenges may arise due to limited access to neonatal intensive care units.

#### Screening of Study Population for Eligibility

The study population was screened based on the following criteria: (1) patients who met the criteria of at least 1 of the 3 screening checklists, which were based on admission diagnosis and presenting signs and symptoms (refer to the “Data Collection Tools and Procedures” section and Multimedia Appendix 2 for the checklist); (2) confirmation or suspicion of 1 or more study outcomes by the treating team; and (3) absence of any exclusion criteria.

**Figure 3:**
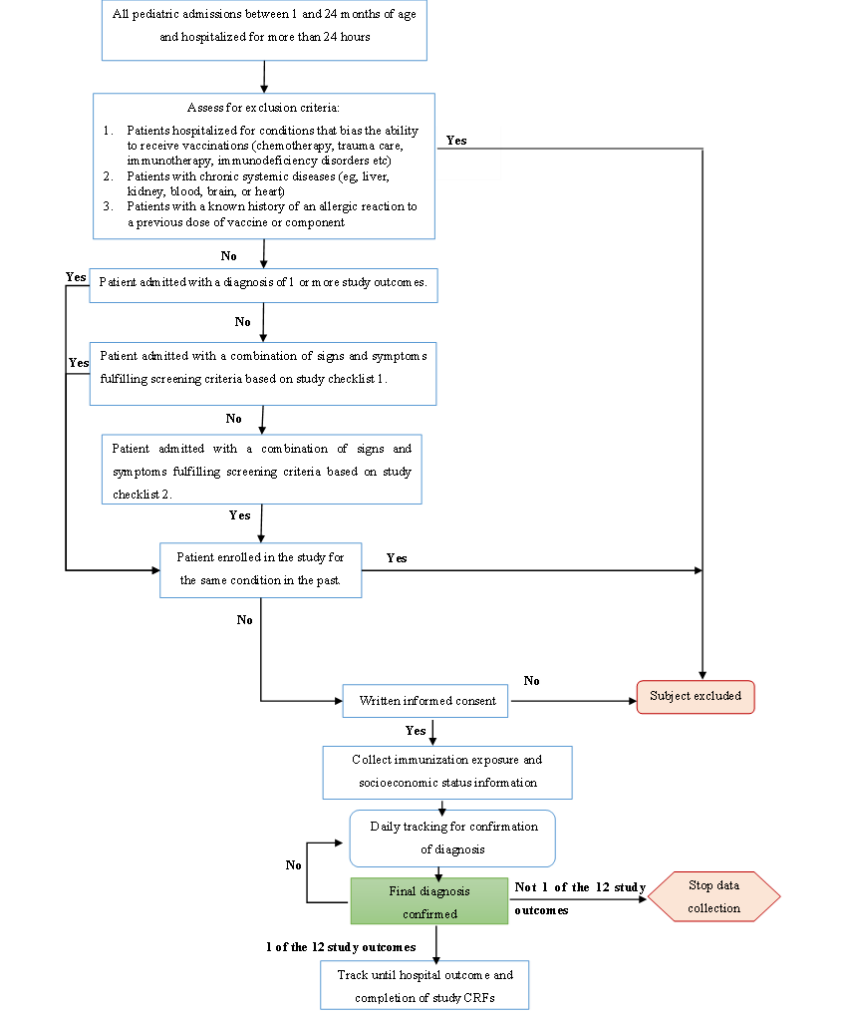
Systematic overview of patient screening, recruitment, and case confirmation processes. CRF: case report form.

The exclusion criteria were (1) diagnosis of a condition that could bias the participant’s ability to receive vaccines, such as immunodeficiency disorders; (2) undergoing therapies that precluded vaccination, for example, chemotherapy or immunotherapy; (3) diagnosis of a chronic systemic disease (eg, liver, kidney, blood, brain, or heart disease); (4) a known history of allergic reaction or anaphylaxis to a previous dose of vaccine or its components; and (5) prior recruitment in the study for the same condition (refer to Figure 3).

#### Selection of Study Outcomes

To broaden the scope of analytical designs for investigating vaccine-event associations, 2 categories of study outcomes were selected: 8 case conditions with published literature supporting their temporal association with 1 or more vaccines, and 4 control conditions with established infectious etiologies and no known link to vaccination [22]. These 12 conditions were highly likely to result in hospitalization, and consistent diagnostic criteria and case definitions could be applied. Table 1 lists the case and control outcomes selected for surveillance in the study.

**Table 1:** List of adverse pediatric study outcomes (case and control conditions) selected for surveillance.

Case conditions: outcomes with prior evidence establishing a temporal association with 1 or multiple vaccinations	Control conditions: outcomes with established etiology and no known association with any vaccinations
Acute demyelinating encephalomyelitis	Dengue
Aseptic meningitis	Malaria
Anaphylaxis	Sepsis
Guillain-Barré syndrome	Urinary tract infection
Intussusception	N/A^a^
Kawasaki disease	N/A
Seizure	N/A
Thrombocytopenia	N/A

^a^N/A: not applicable.

#### Standardizing Case Definitions for Assessing the Level of Diagnostic Certainty

We used the Brighton Collaboration standardized case definitions [23] to assess the level of diagnostic certainty for acute demyelinating encephalomyelitis [24], aseptic meningitis [25], anaphylaxis [26], Guillain-Barré syndrome [27], intussusception [28], Kawasaki disease [29], seizure [30], and thrombocytopenia [31].

The methods developed by the Brighton Collaboration [32] were also adapted to establish accepted case definitions and to ascertain levels of diagnostic certainty for the control outcomes. Dedicated working groups, led by subject-matter experts—all members of the TAG—reviewed published and unpublished literature. The working groups then categorized levels of diagnostic certainty with decreasing specificity and increasing sensitivity. Multimedia Appendix 3 specifies the standardized case definitions developed for the control conditions.

#### Data Collection Tools and Procedures

Data were collected in 2 modes during the study period: paper CRFs from the start of the study to August 2019, and e-CRFs from September 2019 to site closure in March 2020. Initially, paper CRFs were used to ensure uniformity in study processes across all sites, especially in settings with limited digital infrastructure. Furthermore, at the time of study initiation, available electronic data collection applications did not accommodate the complex screening, recruitment, and case confirmation criteria used in this study. Paper CRFs were submitted monthly by sites and, after review and clarification, were digitized using the customized SOMAARTH-I system, which featured built-in double data entry and quality assurance mechanisms [33]. As the study progressed, e-CRFs were deployed to improve data completeness, timeliness, and accuracy, and to facilitate real-time monitoring. To support this, a customized SOMAARTH-III application [34] was developed, featuring built-in validity checks, automated skip patterns, and form activation to digitally replicate the study process. We pilot-tested both the paper and e-CRFs, along with the standardized case definitions for case and control conditions (described above), at 2 sites in New Delhi over a 1-week period. We trained all sites to use SOMAARTH-III during a 2-day workshop in August 2019. Following the training, sites completed a simulation exercise using dummy patient records before receiving clearance to begin electronic data collection.

Figure 3 provides a systematic overview of the participant screening, recruitment, and case confirmation processes in the study. Potential study participants were identified through a daily review of hospital registers and admission records to capture all children aged 1-24 months hospitalized within the previous 24 hours. Upon recruitment, information on socioeconomic status and immunization exposure was collected. The treating team and patient medical records were then consulted to assess the applicability of exclusion criteria. To minimize missed outcomes, 3 screening checklists with increasing sensitivity were developed: the first identified participants diagnosed with or suspected of having study outcomes in admission records, while the subsequent 2 screened for signs and symptoms associated with the study outcomes (refer to Multimedia Appendix 2).

Regardless of immunization exposure status, all participants meeting any of the 3 checklist criteria were eligible for recruitment, pending properly administered and signed informed consent from their primary caregivers.

We considered only documented sources of immunization status—such as mother-child protection cards and immunization registers—as acceptable, due to concerns about the validity of recall-based methods. We aimed to ascertain exposure status as comprehensively as possible, including active outreach to catchment area immunization centers and postdischarge follow-up of primary caregivers of recruited participants.

Concurrently, all recruited participants were tracked daily for diagnosis confirmation, and clinical information was completed once the treating team confirmed a study outcome as the final (primary or secondary) diagnosis. We followed up with recruited participants until the end of their admission—whether death, discharge, or leaving against medical advice—to complete any pending study CRFs.

We followed up with patients after discharge to (1) obtain immunization exposure source documents if these could not be collected during hospitalization; and (2) ascertain the monophasic nature of illness for acute demyelinating encephalomyelitis [24] and Guillain-Barré syndrome [27], as required by their respective standardized case definitions.

#### Data Monitoring and Quality Assurance

We developed a data management plan guided by study policies covering data collection, monitoring, quality assurance, sharing, archival, curation, and dissemination.

The central team remotely reviewed incoming data using structured templates to assess completeness, quality, and adherence to the study protocol and procedures. Any discrepancies were addressed through monthly teleconferences with site teams.

Regular monitoring helped identify sites submitting erroneous, incomplete, or delayed data, enabling targeted support. In the first year, 5 sites were selected for quality assurance visits; in the second year, all sites were visited. During these visits, a team comprising members of the central team and TAG completed a structured questionnaire to assess data quality, evaluate the risk of missing study outcomes, identify challenges and facilitators related to project implementation, and provide recommendations on whether data collection should continue at each site based on their observations. In its June 2018 meeting, the study TAG reviewed findings from the visits and recommended discontinuing 1 site due to significant operational challenges and concerns about the timeliness and completeness of its study data.

#### Sample Size Determination

No sample size calculations were required to address the first 3 objectives; all age-eligible participants diagnosed with 1 or more study outcomes were eligible, pending fulfillment of the study inclusion and exclusion criteria as described in Figure 3. For objective 4, that is, hypothesis testing, we estimated sample size requirements for an unmatched case-control study as an alternative to case-only analytical designs, which are typically more efficient and require smaller sample sizes [35]. Assuming an exposure rate of 20% among controls, a minimum odds ratio of 1.3, 20% incomplete data, and a case-to-control ratio between 1:2 and 1:4, we calculated a required sample size of 3700-5040 participants to test hypothesized vaccine-event associations with 95% confidence and 80% power [36] (see Table 2). Based on pediatric admission data submitted during site screening and the expected prevalence of case and control conditions, we estimated that this sample size (for a 1:2 case-to-control ratio) would be reached in approximately 2.5 years.

**Table 2:** Sample size required for an unmatched case-control study assuming 20% exposure in controls, minimum odds ratio of 1.3, and adding 20% incomplete or missing data for a range of case-to-control ratios at 95% confidence and 80% power.

Categories	Case-to-control ratio			
	1:1, n	1:2, n	1:3, n	1:4, n
Sample size for cases	1339	1016	899	840
Sample size for controls	1339	2032	2695	3358
Total sample size	2798	3048	3594	4198
Projected sample size including incomplete/missing data (20%)	3360	3700	4320	5040

#### Statistical Analysis

For objectives 1, 2, and 3, we analyzed data cumulatively by health outcome using Stata version 15.1 (StataCorp) [37]. Results were stratified by geographic zone. We calculated all 95% CIs using the Clopper-Pearson method [38]. For objective 1, we estimated outcome rates per 100,000 hospitalized children aged 1-24 months per year, with 95% CIs, for each outcome. For objective 2o, we also stratified the cumulative and zone-wise outcome rates by climatological seasons in India, as defined by the Indian Meteorological Department [39]. We calculated the sex and age distribution (with 95% CIs) and the median age (IQR) for all study outcomes. Additionally, we calculated wealth index scores for each participant using a previously standardized method [40]. Based on the calculated wealth score, we classified each case into wealth quintiles using cut-offs from the National Family Health Survey-4 dataset [41]. We calculated the proportion (with 95% CIs) of confirmed outcomes with documented vaccine exposure information. For objective 3, we calculated the proportion of outcomes meeting standardized case definitions stratified by level of diagnostic certainty, as well as reasons for nonclassification to the lowest diagnostic certainty level.

For objective 4, we developed a decision framework to select appropriate primary and secondary analytic designs for each vaccine-event pair by considering relevant exposures (vaccine types), the number of participants recruited, the type of onset (well-defined or insidious), and the expected duration of the risk interval (long or short) for each case outcome based on prior published literature. For case-control analyses, we excluded combinations that tended to co-occur—for example, thrombocytopenia with dengue. The date of hospital admission was used as a proxy for the event date. Sensitivity analyses were conducted based on the date of symptom onset (as recorded in patient medical records), vaccine dose, level of diagnostic certainty for study outcomes, and varying risk intervals.

## Results

### Site Characteristics

The final network included 15 public tertiary care and 7 private hospitals across 17 states and 1 union territory in India. Of the 7 private facilities, 4 were located in the south zone. Two study sites either could not be activated or were closed after a limited recruitment period (see Figure 1). Wide variations were observed in pediatric and age-eligible admissions across sites: the largest site (Government Medical College, Trivandrum) admitted 15,527 children, while the smallest site (Assam Medical College, Dibrugarh) admitted 651 children aged 1-24 months during the study period. Data collection at some sites was disrupted due to local operational challenges. Table 3 describes the characteristics of participating sites, including their geographic distribution, pediatric patient load, patient record–keeping mechanisms, and the number of months of data collected.

**Table 3:** Study site characteristics.

Zone	State/union territories	Site name	Type of facility	Format in which patient case records are maintained	Number of pediatric admissions of children between 1 and 24 months of age, n	Number of months of data collection, n
North	Punjab	Christian Medical College, Ludhiana	Private	Paper	1304	29
North	New Delhi	Lady Hardinge Medical College	Public	Paper	8709	29
North	New Delhi	Maulana Azad Medical College^a,b^	Public	Paper	5849	26
North	Haryana	Pandit Bhagwat Dayal Sharma Post Graduate Institute of Medical Sciences, Rohtak	Public	Paper	8861	29
North	Jammu & Kashmir	Sher-e-Kashmir Institute of Medical Sciences^c^	Public	Paper	1593	27
East	Tripura	Agartala Government Medical College	Public	Paper	3271	29
East	Assam	AMC D^d^	Public	Paper	4069	26
East	Bihar	Indira Gandhi Institute of Medical Sciences, Patna	Public	Paper	651	29
East	Odisha	Institute of Medical Sciences & Sum Hospital	Private	Paper	2316	29
East	West Bengal	Post-Graduate Medical Education and Research and Seth Sukhlal Karnani Memorial Hospital, Kolkata^e^	Public	Paper	2496	22
East	Meghalaya	North Eastern Indira Gandhi Regional Institute of Health and Medical Sciences^f^	Public	Paper	601	27
East	Jharkhand	Rajendra Institute of Medical Sciences	Public	Paper	3167	29
West	Madhya Pradesh	Choithram Hospitals & Research Center^a^	Private	Electronic	995	28
West	Maharashtra	Government Medical College Nagpur	Public	Paper	3122	29
West	Maharashtra	Grant Government Medical College & Sir J. J. Group of Hospitals, Mumbai^a,g^	Public	Paper	2230	26
West	Gujarat	Shri M. P. Shah Medical College^a^	Public	Paper	8652	29
South	Andhra Pradesh	Assam Medical College, Dibrugarh	Public	Paper	4759	29
South	Kerala	Government Medical College, Thiruvananthapuram	Public	Paper	15527	29
South	Karnataka	Kasturba Medical College	Private	Paper	4736	29
South	Tamil Nadu	Mehta’s Hospital	Private	Paper	2821	29
South	Tamil Nadu	PSG Institute of Medical Sciences and Research, Coimbatore	Private	Electronic	2183	29
South	Telangana	Rainbow Children’s Hospital	Private	Electronic	2235	29

^a^These 2 sites initiated data collection in December 2017.

^b^Data missing for June and August 2019 due to loss during the transition to electronic data collection.

^c^Data collection was interrupted in August and September 2019, due to government-mandated restrictions, which prevented the research staff from accessing the site.

^d^This site initiated data collection in February 2018.

^e^This site initiated data collection in April 2018. Further, data collection was interrupted in December 2018 due to staff turnover.

^f^This site initiated data collection in January 2018.

^g^No data were collected at this site for November and December 2018 due to staff turnover.

### Study Population and Outcomes

**Figure 4:**
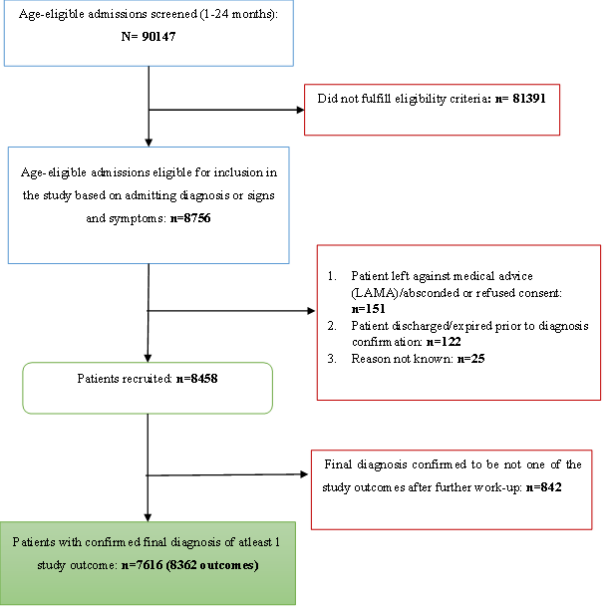
Subjects screened, suspected, recruited, and confirmed cumulatively by Multicenter Active AEFI Surveillance System (MAASS) network sites.

Over the 29-month study period from November 1, 2017, to March 20, 2020, the network admitted 90,147 children aged 1-24 months. Of these age-eligible admissions, 8803 (9.77%) met screening criteria for recruitment, and a final diagnosis was confirmed in 7616 participants. These 7616 participants collectively contributed 8362 confirmed outcomes. Of the 7616 participants, 6959 (91.37%) contributed to only 1 outcome, while the remaining 657 (8.63%) contributed to multiple outcomes. Figure 4 presents the total number of participants and outcomes screened, suspected, recruited, and confirmed. The screening algorithm, based on admission diagnoses and presenting signs and symptoms, identified study outcomes in 7616 of the 8803 suspected participants (86.52%). Immunization documents were available for 6875 of the 8362 participants (82.21%) with confirmed outcomes.

### Data Characteristics

Data timeliness and completeness varied significantly across sites, influenced by the mode, complexity, and type of data collected. The timeliness of data submission was especially affected during the paper-based data collection phase, with delays of 2-8 months observed from the site with the heaviest patient load. On the one hand, paper CRFs were more intuitive and beneficial for sites with initially disrupted internet access; on the other hand, they posed significant challenges in study monitoring, as well as in data storage, transcription, and archival. The transition from paper to electronic data collection optimized many aspects, including overall data quality, timeliness (enabling real-time entry), completeness (due to mandatory response fields in the software), and the rigor of data monitoring, supported by a weekly feedback system. However, the switch also introduced an unforeseen disadvantage: it eliminated our ability to triangulate data across multiple source documents for verification—for example, comparing age recorded in 3 CRFs (screening form, immunization card, and recruitment CRF). Such discrepancies created challenges in assigning a single definitive value in the final dataset. Age mismatches were observed in over 10% (at least 692 age mismatches out of 6830 confirmed outcomes in the paper dataset) of cases when comparing the 3 forms within the paper dataset. Discrepancies in these mismatches often alerted us to other potential data-entry errors, such as inaccuracies in immunization exposure dates. Similar challenges with source-data mismatches have been reported in previous multicenter studies as a significant barrier to ensuring data quality [19,42]. The use of auto-fill features in the electronic dataset further limited our ability to detect and monitor such source-based data discrepancies. Because of varying capacities in digital data collection, some sites required additional technical support and experienced delays during the initial week of the e-CRF module rollout. Although the paper-based phase lasted nearly 3 times longer than the electronic phase, it was reassuring that the overall pattern of outcome identification remained consistent. We confirmed 6830 outcomes from 70,288 (9.72%) age-eligible participants screened during the paper phase and 1532 outcomes from 19,859 (7.71%) participants during the electronic phase.

Basic demographic data (age and sex) and hospitalization details (date of admission and outcome) were available for all participants, while socioeconomic status information was missing for only 14 of the 7616 participants. By contrast, the completeness of clinical description data varied, depending on the number and complexity of the clinical and diagnostic confirmation criteria. For example, fewer data were missing when assessing simple clinical symptoms such as vomiting in intussusception cases—this symptom could not be evaluated in only 6 of 654 intussusception cases, whereas more responses were missing for criteria requiring complex and invasive diagnostic procedures, such as lumbar punctures to assess pleocytosis in cerebrospinal fluid, which is necessary to confirm aseptic meningitis according to the Brighton Collaboration case definition [25]; this criterion could not be evaluated in 131 of 383 aseptic meningitis cases. A more detailed evaluation of the quality of the reported clinical information will be presented in subsequent manuscripts.

Almost all of the 6875 confirmed outcomes with documented immunization exposure included vaccination dates and the administered antigen(s) (single or combination), although vaccine brand names were not recorded on the immunization cards obtained from public sector hospitals.

### Overall Study Timelines and Milestones

**Figure 5:**
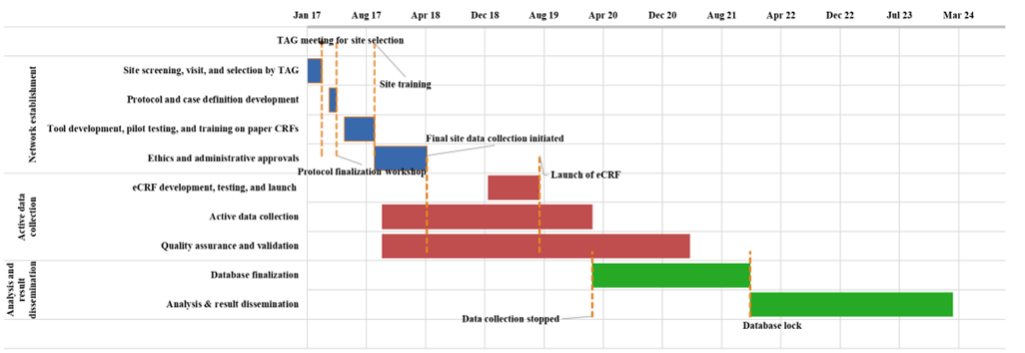
Multicenter Active AEFI Surveillance System (MAASS) study timelines and key milestones.

Figure 5 summarizes the MAASS study timelines and milestones. The study progressed through 3 distinct phases: (1) network establishment phase (January to November 2017), which included site selection, protocol and study tool finalization, as well as ethics and administrative approvals; (2) active data collection phase (November 2017 to March 2020), which involved the development and deployment of electronic data collection systems; and (3) analysis and result dissemination phase (March 2020 to March 2024), which included transcription of paper CRF data, database finalization, analysis for all 4 objectives, and preparation of study reports and manuscripts.

## Discussion

### Expected Findings

This manuscript describes the operational framework for establishing India’s first geographically and socioculturally representative active surveillance network to monitor pediatric health outcomes and vaccine exposure.

The framework’s feasibility was demonstrated through successful consensus on governance and procedures, the establishment of a 22-hospital sentinel network, standardized protocols and data collection methods, deployment of electronic data systems with a unified database, rigorous quality control measures, and the capacity to link health outcomes to immunization records in a large study population for robust safety analyses. While the MAASS network demonstrated feasibility, the long-term sustainability of such networks depends on addressing additional challenges, including securing sustained funding for staff training and retention, site maintenance, technical support, and ensuring ongoing commitment from investigators and institutions. Variability in data quality across sites necessitated targeted site support, standardized training programs, and periodic quality audits of participating centers.

The initial results from this initiative highlight the potential of hospital-based sentinel surveillance for actively monitoring pediatric outcomes and immunization exposure in resource-limited settings, including LMICs. The network generated a substantial database of 8362 outcomes and documented vaccine exposure for 6875 (82.22%) cases. The size and representativeness of the sentinel hospital network and the population, combined with the established operational framework, provide a strong foundation for future developments and offer a valuable resource for conducting subsequent studies without the need to start from scratch. The variability observed in the database—across data collection modes, complexity, and source documentation—offered valuable insights for enhancing data quality in vaccine pharmacovigilance. The MAASS network has the potential to transform the national surveillance program by complementing existing passive surveillance systems and strengthening the evidence base for the safety of both traditional and new vaccines, thereby bolstering public confidence.

Several operational challenges were encountered in establishing the MAASS study network, offering valuable lessons for future development and for other resource-limited settings aiming to build similar capacity. Complex ethics and administrative requirements delayed the start of data collection by an average of 3.5 months, with the final site taking up to 7 months to obtain approval. National ethics institutions need to develop guidelines specifically for multicentric network studies to streamline and accelerate approval processes, incorporating mechanisms that ensure consistency in core study protocols [15]. This is particularly important to facilitate international scientific collaboration in today’s increasingly global public health landscape [5].

Paper-based data collection was resource-intensive and delayed error detection, with quality assurance hindered by a 1-month lag between data collection and review. The introduction of e-CRFs significantly reduced this time lag, minimized errors from repeated data entry, improved overall data quality, and helped ensure closer adherence to the study protocol. Previous experiences comparing paper-based and electronic data collection have demonstrated similar challenges and their resolution [19]. Digital tools improve efficiency by embedding accountability and validation checks and help reduce long-term costs. In our study, limited access to reliable internet connectivity, especially at remote sites, posed a significant challenge to the timely submission of electronic data. This challenge was addressed by incorporating offline data entry capabilities within the software. Moving forward, allocating additional resources to ensure adequate internet access will be crucial for establishing rapid and responsive pharmacovigilance networks, even in resource-limited settings. Furthermore, providing dedicated training to study staff on digital data collection procedures at the outset of the study—including hands-on simulation exercises—can help mitigate challenges encountered during the transition from paper-based CRFs to e-CRFs. Significant operational challenges resulted in temporary disruptions to data collection at 4 network sites (Table 3). At 2 of these sites, data collection resumed only after new or additional research staff were allocated and trained. At another site, operations could recommence only after access to the facility was restored.

Despite these challenges, the MAASS network demonstrated a scalable model that can be adapted to other resource-constrained settings, particularly in light of the rapidly evolving immunization landscape. Passive surveillance alone is often insufficient to provide the comprehensive data needed to evaluate newly introduced vaccines targeting broader age groups and demographics, or to detect long-term and rare adverse events. Integrating both public and private sector health care facilities enables a more comprehensive approach to data collection. Simultaneously, the use of harmonized Brighton Collaboration case definitions and confirmation criteria offers a reliable framework for generating comparable vaccine safety data across diverse geographic settings. The use of a tiered approach to patient screening and outcome confirmation enhances the network’s ability to identify complex health outcomes across sites with varying health infrastructure and clinical practices. Future iterations built on the MAASS network infrastructure could leverage advanced analytic approaches—such as machine learning and artificial intelligence—to automate signal detection across disparate data sources and reduce delays in case ascertainment through natural language processing of clinical notes [43].

### Limitations

As with most hospital-based studies, ours lacked well-defined catchment populations, limiting the ability to generate true population-based estimates [14]. To address this limitation, we used all hospital admissions of children aged 1-24 months during the study period as the denominator, assuming that referral patterns remained relatively consistent for each participating tertiary care facility. Although the selection of hospitals in our study aimed to ensure geographic representativeness, the identification of outcomes and the estimation of hospital outcome rates were influenced by factors such as the type of facility, the availability of infrastructure and diagnostic resources, and the health care–seeking behaviors of the populations served [44]. Expanding the sentinel network in the future may help reduce regional disparities. Depending on a country’s size and other characteristics, both regional and national network–based background rates—as well as deviations from baseline—can be effectively monitored. Linking this information with vaccine exposure data can enhance the quality of risk assessment studies. As new vaccines are introduced targeting broader age groups and geographic areas, establishing community-based background rates will be essential for verifying potential vaccine safety signals.

The study population was restricted to children aged 1-24 months, thereby excluding data on neonatal immunizations and immunizations administered later in childhood. Future studies may consider adjusting or expanding the age range to capture a broader spectrum of immunization-related outcomes. Additionally, the inclusion criterion of hospital admissions exceeding 24 hours excluded cases managed entirely in emergency care settings. For instance, instances of anaphylaxis treated and discharged directly from the emergency department were not captured in this study.

Over 90,000 children were hospitalized across participating study sites during the study period. The screening algorithm identified 8803 of the 90,147 (9.77%) children as suspected cases of specific outcome conditions, of which 7616 (86.52%) were subsequently confirmed. However, it remains unknown how many true outcome cases may have been missed among the nearly 80,000 admissions not flagged by the screening tool. This limitation could lead to an underestimation of the true background rates generated by the study.

Variability in the completeness of clinical information—driven by the complexity and type of data collected—and in vaccine exposure data may impact 2 critical aspects of vaccine safety monitoring: (1) the comparability of the level of diagnostic certainty across study outcomes, and (2) the ability to assess associations between adverse outcomes and specific vaccine types or brands. Future safety studies could benefit from more rigorous efforts to collect detailed vaccine exposure information—including brand, batch number, and timing of vaccination—potentially by integrating with administrative immunization data from existing national health management information systems.

Finally, our study highlighted the need to develop additional case definitions and data collection guidelines for a reference set of clinical outcomes (controls) with specific etiologies known not to be associated with vaccine exposure. The standardized case definitions developed for the controls should undergo further field testing before wider application. Although beyond the scope of this study, the sensitivity, specificity, positive predictive value, and negative predictive value of all standardized case definitions related to vaccine safety monitoring need rigorous evaluation and contextual adaptation to ensure precise and reliable outcome identification.

### Comparison With Prior Work

The need to establish global frameworks for vaccine safety monitoring is well recognized [11,12]. While adequate methods and methodological frameworks have been developed in some regions, international efforts toward harmonizing vaccine safety assessment are ongoing [45,46]. Efforts in LMICs remain limited, highlighting the need for innovative approaches. Our study builds upon methodological insights from high-income settings as well as selected pilot experiences in LMICs. Previous hospital-based active sentinel surveillance studies for vaccine safety monitoring in LMICs have been limited by the scope of outcomes and vaccine exposures assessed. The Global Vaccine Safety Multi-Country Collaboration study assessed the risk of immune thrombocytopenia and aseptic meningitis following measles-mumps–containing vaccines across 26 sites in 11 LMICs and 5 high-income countries [47]. In India, 2 separate tertiary care hospital–based network studies have evaluated the risk of intussusception following the introduction of the rotavirus vaccine over the past decade [20,48]. All 3 studies used self-controlled case series as the primary analytical design to estimate the relative incidence of selected outcomes following vaccination, using predefined risk intervals [47-49]. In Singapore, a single hospital-based active surveillance study identified a fivefold increase in postvaccination Bacillus Calmette-Guérin vaccine–associated lymphadenitis rates, which prompted further investigation of this safety signal [50]. All studies reported similar operational challenges, notably significant delays due to ethics and regulatory approvals from multiple authorities [19,50,51]. Future research will benefit from streamlining ethics and regulatory processes at global, regional, and national levels to facilitate multicenter studies. Collectively, these studies demonstrate the applicability of hospital-based active sentinel surveillance to address a broad spectrum of postlicensure vaccine pharmacovigilance challenges. These include establishing baseline outcome rates, characterizing the clinical and sociodemographic profiles of rare adverse events, detecting previously unrecognized safety signals, and investigating associations between specific vaccines and adverse outcomes. While the MAASS network offers a scalable model for active vaccine safety surveillance in resource-constrained settings, its long-term sustainability depends on implementing digital solutions tailored to local infrastructure, providing targeted site support, and increasing advocacy for domestic funding of such networks. With the growing ability to leverage additional data sources, such as social media and solicited reports, active vaccine safety surveillance systems can both detect previously unreported safety signals (eg, via cohort event monitoring) and investigate or verify reported signals using hypothesis-testing study designs [52].

### Conclusions

The MAASS study, unprecedented in scale, provides a scalable blueprint for active vaccine safety surveillance in resource-limited settings. The adoption of globally standardized case definitions, along with the development of definitions for reference control outcomes, has strengthened the capacity of participating sites and encouraged improved documentation of immunization exposure and diagnostic evaluations in routine patient care. Beyond India, the MAASS framework tackles key questions about the feasibility of active surveillance networks in resource-constrained settings and offers a template to advance global vaccine safety equity. Future networks should focus on long-term sustainability; harness rapidly evolving digital health infrastructure; and broaden the range of conditions, vaccines, and populations assessed to generate robust vaccine safety data from LMICs. The study addresses critical gaps in understanding rare and complex pediatric health outcomes and bolsters ongoing efforts to enhance vaccine pharmacovigilance capacity in India.

## Appendix

Multimedia Appendix 1 Site screening and selection questionnaire.

Multimedia Appendix 2 Outcome screening checklists.

Multimedia Appendix 3 Standardized case definitions for control conditions.

## Abbreviations

AEFI: adverse events following immunization
CRF: case report form
e-CRF: electronic case report form
INCLEN: International Clinical Epidemiology Network
LMIC: low- and middle-income country
MAASS: Multicenter Active AEFI Surveillance System
TAG: technical advisory group
WHO: World Health Organization

## Data Availability

All data are available from the corresponding author of this study. Given the sensitive nature of patient-linked information collected in this study, fully anonymized datasets are available from the corresponding author upon reasonable request, in alignment with India’s national ethical guidelines for biomedical research [21].
