# Supplementary material for: Establishing a Multicenter Active Adverse Events Following Immunization Sentinel Surveillance Network Across 22 Tertiary Care Hospitals in India: Protocol for a Prospective Observational Study
Source: JMIR Res Protoc. 2025 Aug 8;14:e64050. doi: 10.2196/64050 (PMC12374136; doi:10.2196/64050)
Supplement: Multimedia Appendix 1 [file resprot_v14i1e64050_app1.pdf]

Appendix material A1: Site screening and selection questionnaire

Screening questionnaire

A. General information (Please fill the response)

(Please provide information about the concerned departments in the hospital)

Sl No	Item/ Question	Paediatrics	Paediatric Surgery	Microbiology	Radiology	Pathology	Forensic
1	Number of beds						
2	Number of units						
3	Number of Specialists (Teaching faculty/ non-teaching specialists)						
4	Academic Residents (MD/DNB/DM/ MCH/ Fellows)						
5	Non-academic Residents						
6	Total number of paediatric admissions in last 1 year						

7	Number of children less than 2 yrs of age admitted in last 1 year						

B. Clinical services

Sl no	Questions/ Items<br><br>*(Please mark √in appropriate box; mark as many apply)*	Response
**1**	**Availability of round the clock services**	
1.1	Is the paediatric service available round the clock in the hospital?	
1.2	Is the paediatric surgery service available round the clock in the hospital?	
1.4	Is the Emergency Service available round the clock in the hospital?	
1.6	What is the process(es) for admission to the hospital/departments?	
	a) Through OPD	
	b) Through emergency department/ casualty	
	c) Transfer from other departments	
	d) Others<br><br>(Please specify…………………………………………………………)	
**2**	**Is there a separate ward / unit for emergency admission of children?**	

2.1	If yes, how many beds are earmarked for children?	
2.2	What is the maximum duration of hospitalization in the emergency ward/unit before transfer to specific ward/unit?	
2.3	Who looks after the clinical care of the patients in the emergency unit/ward?	
**3**	**Are the following paediatric sub-specialty services/units available**	
3.1	Paediatric neurology	
3.2	Paediatric gastroenterology	
3.3	Paediatric nephrology	
3.4	Paediatric cardiology	
3.5	Paediatric haematology	
3.6	Any other (Please specify……………………………….)	
**4**	**Do you use any specific template/format for case clinical history record and clinical progress documentation?**	
4.1	Does the clinical history include collection of immunization details?	
4.2	If yes, how is the immunization history captured?	
a	In summary	
b	Vaccine/antigen wise detailed immunization history	
**5**	**Is there a provision of emergency laboratory services for diagnostics to be performed beyond routine hours in the ward/unit?**	

5.1	If yes, what all tests are being done/can be done in the laboratory?<br><br>(Pease specify)	
**6**	**Does the paediatric department have access to the following:**	
6.1	Computer/Laptop	
6.2	Scanner	
6.3	Internet connectivity	
**7**	**Please specify the kind of internet connection available (Like LAN, Data card, WiFi, smart phone etc.)**	
**8**	**Is there a space available to house the research staff to be engaged in the study? (2 research staff)**	

C. Diagnostic services

Sl no	Questions/ Items	Response
**1**	**Availability of the services**	
1.1	Are the following services available at the hospital:	
	**Biochemistry/biochemical laboratory**	
	a) Biochemistry	
	b) Immunology/Serology	
	**i) Microbiology laboratory services**	

	a) Bacteriology	
	b)Virology	
	c) Parasitology	
	d) Fungus/ other microbes	
	e) Immunology/Serology	
	**ii) Pathology laboratory services**	
	a) Pathology/Histopathology	
	b) Cytopathology	
	c) Immunopathology	
	d) Forensic Pathology including autopsies	
	**iii) Radiology services**	
	a) Ultrasound	
	b) X rays (specify if digital X-ray available)	
	c) MRI scanning	
	d) CT scanning	
	**iv) Nuclear medicine diagnostic laboratory**	
	**v) Electro/Neurophysiology diagnostic laboratory**	
	a) EEG	
	b) NCV/EMG	

**2**	**If the above diagnostic facilities are not available in the hospital, does the hospital have a standard referral laboratory for these?**	
2.1	Microbiology laboratory services	
2.2	Pathology laboratory services	
2.3	Radiology laboratory services	
2.4	Nuclear medicine diagnostic laboratory	
2.5	Electro/Neurophysiology diagnostic laboratory	
**3**	**Does the hospital have storage facility for biological samples?**	
3.1	-20° C	
3.2	-70/80° C	
**4**	**Is there any laboratory in the ward/ emergency laboratory with access beyond the routine hours?** (like blood smear, CSF cell count)	
**5**	**Do the radiological instruments/machines have facility for digital storage/image archival?**	
5.1	If yes, for how long are the digital images of the ultrasound tests stored/archived?	
5.2	Are these digital images retrievable?	

D. Vaccination services and record keeping:

Assessing vaccine exposure in hospitalized children is a key component of the study. The following questions seek to understand mechanisms for recording and retrieving vaccine

**1**	**Do you administer vaccinations in the hospital?**	

**2**	**Are vaccines that are not included in UIP made available on additional charge?**	
**3**	**What is the average number of vaccines administered annually?**	
**4**	**How is the record for vaccinations maintained at the hospital?**	
4.1	Computerized/ electronic records	
4.2	Manual records/listed in register	
4.3	Any other method<br>(Specify ……………………………………………………….)	
**5**	**Are vaccination records retrievable?**	
**6**	**How are Adverse Events Following Immunization (AEFIs) recorded at the hospital?**	
**7**	**Does the institute (pharmacology department) have an Adverse Drug Reaction Monitoring Center?**	

E. Medical Record Archival System

The project requires screening/review of the hospitalized children less than two years of age to capture clinical conditions/events of interest. Thus, listing and retrieval of medical records are very essential. The following questions/items focus on the admission, medical record archival and retrieval process. This will allow planning the case record tracking and retrieval at the institution.

Sl no	Questions/ Items<br><br>*(Please mark √in appropriate box; mark as many apply)*	Response		
		**Yes**	**No**	
**1**	**Is the admission registration for hospital centralized?**			***If NO, skip to Q 3***
**2**	**If yes, what is the system of admission registration?**			
2.1	Computerized/ electronic admission registration			
2.2	Manual admission registration			
2.3	Any other method (Specify …………………………………...)			
**3**	**Does the institution have a centralized medical record index/ Master Patient Index/ Centralized Admission Register?**			***If NO, skip to Q 5***
**4**	**What is the system for recording discharges in the hospital?**			
4.1	Computerized/ electronic			
4.2	Manual			
4.3	Any other method<br><br>(Specify ……………………………………………………….)			
**5**	**Are records for admission and discharge retrievable?**			
**6**	**How are patient records maintained across multiple units/wards?**			
6.1	Every unit maintains its own register			
6.2	Combined/ single register for all units			

6.3	Registers are maintained floor wise			
6.4	Any other method (specify……………………………………)			
6.5	Specify if patient records are maintained digitally or manually?			
**7**	**What information can be retrieved from the ward/unit admission register?**			
7.1	Admission number			
7.2	Date of admission			
7.3	Department of admission			
7.4	Name			
7.5	Age			
7.6	Sex			
7.7	Diagnosis at admission			
7.8	Final diagnosis			
7.9	Outcome (discharge/ death/LAMA)			
7.10	Date of outcome			
**8**	**How are case-sheets (hard copies) stored/ archived in your hospital/institution for in-patients cases after discharge/death/LAMA**			
8.1	Admission number wise			
8.2	Department wise			

8.3	Date of admission wise			
8.4	Date of discharge wise			
8.5	Any other method (specify……………………………………)			
**9**	**How are in-patient case records indexed/ listed in the medical record sector/department?**			
9.1	Computerized/ electronic listing			
9.2	Manual listing			
9.3	Bundled/packed as per date/month of admission/discharge			
9.4	Any other method (specify……………..……………………..)			
9.5	Not listed			
**13**	**Are the death cases archived separately?**			
**14**	**Does the institute summarize the medical records of hospitalized patients periodically according to any classification system** (ICD 10 or any other)?			
**15**	**Is there a mortuary in the hospital?**			
**16**	**Are autopsies performed at the hospital?**			

F. Institute authority approvals

The project requires access to the case-sheets of children with clinical conditions/events of interest, admitted to your hospital and collection of clinical and vaccine exposure details about them. Please inform about the approvals from the institute authority(ies) to be obtained for the same.

Sl no	Questions/ Items	
**1**	**What all permissions from institute/department authorities are needed and the time needed for the same?**	
	Authority	Time needed
1.1		
1.2		
1.3		
1.4		
**2**	**How frequently (in months) does the institute ethics committee meets?**	Monthly
**3**	**List key challenges anticipated in this process?**	

**4**	**How can these challenges be addressed?**
**5**	**Any other specific comments/ suggestions?**

G. Investigating Team Members

Please inform about potential investigating team members from the institute along with their affiliations and contact information.

	Name	Department	Designation	Address details (including email and phone)
1				
2				

3				
4				

1 *Site selection visit questionnaire*

A. General information

Sl.No.	Item	Response
1	Name of the assessor	
2	Institution visited	
3	Date(s) of visit	

B. Clinical Department/Ward

- ***PEDIATRIC MEDICINE***

(Please visit the paediatric medicine ward and review appropriate records for answering the following section, with the help of the study PI)

Key Informant(s) *(Site investigator/Paediatrician, Paediatric nurse, etc.)*

	Department	Name	Designation	Brief observation (positive/neutral/negative)
1	Ped Medicine			
2	Ped Medicine			
3	Ped Medicine			
4	Ped Medicine			

Sl no	Item/ Question	Response
1	No. of paediatric medicine admissions in last 1 week<br>• In ward<br>• In the ICU*<br>• Paediatric ICU*<br>• Neonatal ICU*<br>*as applicable	
2	No. of paediatric medicine discharges in last 1 week<br>• In ward<br>• In the ICU*<br>• Paediatric ICU*<br>• Neonatal ICU*<br>*as applicable	
3	Please check the admission register in the ward for:<br>a) Status of update/entry of fresh admission (completeness of entry for recent admissions on same day)	Yes ☐<br>No ☐
	b) Availability of admission diagnosis for the admitted cases	Yes ☐<br>No ☐
	c) Availability of final diagnosis for the discharged cases	Yes ☐<br>No ☐
4	Please review case records of 5**admitted** children (<2yrs) to document the following **(yes/no), please also comment for any specific observation**	

Item	Case 1	Case 2	Case 3	Case 4	Case 5
a) Structured case record format (specific template used)	Yes ☐<br>No ☐	Yes ☐<br>No ☐	Yes ☐<br>No ☐	Yes ☐<br>No ☐	Yes ☐<br>No ☐
b) Admission diagnosis	Yes ☐<br>No ☐	Yes ☐<br>No ☐	Yes ☐<br>No ☐	Yes ☐<br>No ☐	Yes ☐<br>No ☐
c) Immunization history - with details of vaccines received	Yes ☐<br>No ☐	Yes ☐<br>No ☐	Yes ☐<br>No ☐	Yes ☐<br>No ☐	Yes ☐<br>No ☐
d) Date/age at vaccination for vaccines	Yes ☐<br>No ☐	Yes ☐<br>No ☐	Yes ☐<br>No ☐	Yes ☐<br>No ☐	Yes ☐<br>No ☐
e) Copies of investigation reports attached	Yes ☐<br>No ☐	Yes ☐<br>No ☐	Yes ☐<br>No ☐	Yes ☐<br>No ☐	Yes ☐<br>No ☐

	Specific observations/comments:
5	How long are case sheets of discharged patients stored/ kept in the ward before sending for archiving?
6	Please review some case records of **discharged** children (<2yrs) that are still available in the ward, to document the following **(yes/no):**

a) Outcome noted (Death/Discharge/Left Against Medical Advice)	Yes ☐ No ☐
a) Final diagnosis noted	Yes ☐ No ☐
b) Discharge slip attached	Yes ☐ No ☐
c) Copy of discharge sheet kept in the ward/in the department	Yes ☐ No ☐
d) If yes, where is the file/archival made?	

7	Please comment on the overall quality of the case-records reviewed.<br>*(completeness of history, clarity of documentation for retrieval of information)*
8	Please ask any **10 parents/primary caregivers** of admitted children (<2yrs) for availability of their vaccination card **(Y-yes/ N-no):**

**Item**	**1**	**2**	**3**	**4**	**5**	**6**	**7**	**8**	**9**	**10**
a) Vaccination card available at hospital	Y ☐<br>N ☐	Y ☐<br>N ☐	Y ☐<br>N ☐	Y ☐<br>N ☐	Y ☐<br>N ☐	Y ☐<br>N ☐	Y ☐<br>N ☐	Y ☐<br>N ☐	Y ☐<br>N ☐	Y ☐<br>N ☐
b) Vaccination card available but not at hospital	Y ☐<br>N ☐	Y ☐<br>N ☐	Y ☐<br>N ☐	Y ☐<br>N ☐	Y ☐<br>N ☐	Y ☐<br>N ☐	Y ☐<br>N ☐	Y ☐<br>N ☐	Y ☐<br>N ☐	Y ☐<br>N ☐
c) If not available, how long it may take to get the imm card.										

	d) Vaccination card not available (at all)	Y ☐ N ☐	Y☐ N☐	Y ☐ N ☐	Y☐ N☐	Y ☐ N ☐	Y☐ N☐	Y ☐ N ☐	Y☐ N☐	Y ☐ N ☐	Y☐ N☐

9	Are there any **currently admitted patients** (<2 yrs) diagnosed/have a suspected diagnosis of the following clinical conditions in the **ward as well as the ICU, PICU, NICU**	
	**Condition**	**No. of patients**
	a) Acute Flaccid Paralysis/Acute demyelinating encephalomyelitis/Aseptic meningitis	
	b) Anaphylaxis	
	c) Seizure	
	d) Thrombocytopenia	
	e) Kawasaki's disease	
	f) Sepsis (Total and number where blood culture sent)	
	g) Pyelonephritis (Total and number where blood culture sent)	
	h) Malaria	
	i) Dengue	
	j) Intussusception	
10	Is there any specialty unit in the paediatric medicine ward (like neurology, haematology, etc.)?<br><br>If yes, mention details	
11	*Ask the site investigator:* What proportion of the client at this hospital belong to the following catchment areas (approx):	
	a) Same district	
	b) Same state	
	c) Other state	
12	Any specific comments/observations	

2. PEDIATRIC SURGERY

(Please visit the paediatric surgery ward and review appropriate records for answering the following section, with the help of the study PI)

Key Informant(s)

(Site investigator/Paediatric surgeon, nurse, etc.)

	Department	Name	Designation	Brief observation (positive/neutral/negative)
1	Ped Surgery			
2	Ped Surgery			
3	Ped Surgery			
4	Ped Surgery			

**Sl no**	**Item/ Question**	**Response**
1	No. of paediatric surgery admissions in last 1 week<br>• In ward<br>• In the ICU*<br>• Paediatric ICU*<br>• Neonatal ICU*<br>*as applicable	
2	No. of paediatric surgery discharges in last 1 week<br>• In ward<br>• In the ICU*<br>• Paediatric ICU*<br>• Neonatal ICU*<br>*as applicable	
3	Please check the admission register in the ward for:<br>d) Status of update/entry of fresh admission (completeness of entry for recent admissions on same day)	Yes ☐<br>No ☐
	e) Availability of admission diagnosis for the admitted cases	Yes ☐

<table>
<tr><td></td><td colspan="5"></td><td>No ☐</td></tr>
<tr><td></td><td colspan="5">f) Availability of final diagnosis for the discharged cases</td><td>Yes ☐
No ☐</td></tr>
<tr><td>4</td><td colspan="6">Please review case records of 5admitted children (<2yrs) to document the following (yes/no), please also comment for any specific observation</td></tr>
<tr><td></td><td>Item</td><td>Case 1</td><td>Case 2</td><td>Case 3</td><td>Case 4</td><td>Case 5</td></tr>
<tr><td></td><td>f) Structured case record format (specific template used)</td><td>Yes ☐
No ☐</td><td>Yes ☐
No ☐</td><td>Yes ☐
No ☐</td><td>Yes ☐
No ☐</td><td>Yes ☐
No ☐</td></tr>
<tr><td></td><td>g) Admission diagnosis</td><td>Yes ☐
No ☐</td><td>Yes ☐
No ☐</td><td>Yes ☐
No ☐</td><td>Yes ☐
No ☐</td><td>Yes ☐
No ☐</td></tr>
<tr><td></td><td>h) Immunization history - with details of vaccines received</td><td>Yes ☐
No ☐</td><td>Yes ☐
No ☐</td><td>Yes ☐
No ☐</td><td>Yes ☐
No ☐</td><td>Yes ☐
No ☐</td></tr>
<tr><td></td><td>i) Date/age at vaccination for vaccines</td><td>Yes ☐
No ☐</td><td>Yes ☐
No ☐</td><td>Yes ☐
No ☐</td><td>Yes ☐
No ☐</td><td>Yes ☐
No ☐</td></tr>
<tr><td></td><td>j) Copies of investigation reports attached</td><td>Yes ☐
No ☐</td><td>Yes ☐
No ☐</td><td>Yes ☐
No ☐</td><td>Yes ☐
No ☐</td><td>Yes ☐
No ☐</td></tr>
<tr><td></td><td colspan="6">Specific observation/comments:</td></tr>
<tr><td>5</td><td colspan="6">How long are case sheets of discharged patients stored/ kept in the ward before sending for archiving?</td></tr>
<tr><td>6</td><td colspan="6">Please review some case records of discharged children (<2yrs) that are still available in the ward, to document the following (yes/no):</td></tr>
<tr><td></td><td colspan="5">b) Outcome noted (Death/Discharge/Left Against Medical Advice)</td><td>Yes ☐ No ☐</td></tr>
<tr><td></td><td colspan="5">e) Final diagnosis noted</td><td>Yes ☐ No ☐</td></tr>
<tr><td></td><td colspan="5">f) Discharge slip attached</td><td>Yes ☐ No ☐</td></tr>
</table>

	g) Copy of discharge sheet kept in the ward/in the department	Yes ☐ No ☐
	h) If yes, where is the file/archival made?	
7	Please comment on the overall quality of the case-records reviewed.<br><br>*(completeness of history, clarity of documentation for retrieval of information)*	
8	Please ask any **10 parents/primary caregivers** of admitted children (<2yrs) for availability of their vaccination card **(Y-yes/ N-no):**	

**Item**	**1**	**2**	**3**	**4**	**5**	**6**	**7**	**8**	**9**	**10**
e) Vaccination card available at hospital	Y ☐<br>N ☐	Y ☐<br>N ☐	Y ☐<br>N ☐	Y ☐<br>N ☐	Y ☐<br>N ☐	Y ☐<br>N ☐	Y ☐<br>N ☐	Y ☐<br>N ☐	Y ☐<br>N ☐	Y ☐<br>N ☐
f) Vaccination card available but not at hospital	Y ☐<br>N ☐	Y ☐<br>N ☐	Y ☐<br>N ☐	Y ☐<br>N ☐	Y ☐<br>N ☐	Y ☐<br>N ☐	Y ☐<br>N ☐	Y ☐<br>N ☐	Y ☐<br>N ☐	Y ☐<br>N ☐
g) If not available, how long it may take to get the imm card.										
h) Vaccination card not available (at all)	Y ☐<br>N ☐	Y ☐<br>N ☐	Y ☐<br>N ☐	Y ☐<br>N ☐	Y ☐<br>N ☐	Y ☐<br>N ☐	Y ☐<br>N ☐	Y ☐<br>N ☐	Y ☐<br>N ☐	Y ☐<br>N ☐

9	Are there any **currently admitted patients** (<2 yrs) diagnosed/have a suspected diagnosis of the following clinical conditions **ward as well as the ICU, PICU, NICU**

**Condition**	**No. of patients**
k) Acute Flaccid Paralysis/Acute demyelinating encephalomyelitis/Aseptic meningitis	
l) Anaphylaxis	
m) Seizure	
n) Thrombocytopenia	
o) Kawasaki's disease	

	p) Sepsis (Total and number where blood culture sent)	
	q) Pyelonephritis (Total and number where blood culture sent)	
	r) Malaria	
	s) Dengue	
	t) Intussusception	
9	Is there any specialty unit in the paediatric surgery ward?<br><br>If yes, mention details	
10	Any specific comments/observations	

C. Immunization Services

(A key component of the surveillance network is the ability to retrieve immunization exposure for suspected cases. Please visit the immunization room to observe the next section)

Key Informant(s)

	Department	Name	Designation	Brief observation (positive/neutral/negative)
1				
2				
3				
4				

SI.no	Item/ Question	
1	Are vaccines not covered under Universal Immunization Programme also administered (at additional cost/procured by parents) at the site?	
2	Is an immunization register maintained?	Yes ☐ No ☐
3	If yes, does it capture the following details?	
	a) Name	Yes ☐ No ☐
	b) Age	Yes ☐ No ☐
	c) Sex	Yes ☐ No ☐
	d) Vaccine administered	Yes ☐ No ☐
4	If yes, please comment on the level of detail captured in immunization information records (batch no., lot no., expiry dates etc.):	
5	Any specific comments/observations	

D. Laboratory &Diagnostics Services

(Please document the information about diagnostic services available at the microbiology,/pathology, radio diagnostics, neurology department and meet with relevant officials from these departments)

Key Informant(s)

(Faculty/Technician, etc.)

	Department	Name	Designation	Brief observation (positive/neutral/negative)
1				
2				

3				
4				
5				

Sl no	Item/ Question	
1	What is the blood culture positivity rate (%) (for any organism)? (approx.)	
2	What is the urine culture positivity rate (%) (for any organism)? (approx.)	
3	How easy/complete is the potential retrieval of microbiology/ pathology reports for patients of interest? *[Please check lab registers for level of detail captured like name/Admission no./sample type/date/finding/result etc.) and provide overall feedback below. May collect a blank template of the registers.]*	
3a	Microbiology	
3b	Pathology	
	**RADIODIAGNOSTICS**	
4	How easy/complete is the retrieval and storage of reports of radiological investigations for patients of interest? *(If needed, can the old reports be retrieved from the register for a patient?)*	
	a) X-rays	
	b) Ultrasound reports	
	c) MRI scan reports	
	d) CT-scan reports	
	e) Any other reports	

	**NEUROLOGY**
5	How easy/complete is the retrieval and storage of other specialized investigations (nuclear imaging/ EEG/NCV etc.) reports for patients of interest?<br><br>Need to add if more services are available
6	How are the reports in the above mentioned departments archived and the duration for which they are stored?
7	Any other specific observation/ comment.<br>*(What would be the prime source of reports? Any special approvals needed to access the reports? What are the prerequisites needed to trace the report from above depts.?)*

E. Medical Record Archival System

(Please meet with the MRD in-charge for answering the following questions)

Key Informant(s)

	Department	Name	Designation	Brief observation (positive/neutral/negative)
1				
2				
3				
4				

Sl no	Questions/ Items
1	How frequently are in-patient case-sheets (hard copies) sent to Medical Records Department (MRD) from the wards?
2	How are in-patient case-sheets (hard copies) stored/ archived in MRD? (such as admission date/department/discharge date wise or any other method)
3	How are the in-patient case-sheets (hard copies) indexed/ listed (computerized/manual listing or any other method)?

4	Does the institute/MRD code/summarize the medical records of hospitalized patients periodically according to any classification system (ICD 10 or any other)?
5	How frequently is the classification labelling of cases and entries are made in register/ computer?
6	Is there any policy by the hospital regarding duration of archival/ storage of the in-patient case-sheets (hard copies)?
7	What is the process of retrieval of the case records from the Medical Records Department (permissions required/time needed etc.)?
8	• **Digitization** refers to ‘‘the action or process of digitizing; the conversion of analogue data (esp in later use images, video and text) into digital form.’’<br>• **Digitalization** by contrast, refers to “the adoption or increase in the use of digital or computer technology by an organization, industry, country, etc”<br><br>a) What is the process of digitization in the MRD (completeness, type of documents and till the year)?<br><br>b) Duration of archival

	c) Are archived records linked with investigation/laboratory reports<br><br>d) Extent of digitalization in your institution? (hospital information system)
9	***Question to the MRD in-charge*****:** What are the challenges faced by the MRD in retrieving records for surveillance (time needed/access for research staff/approval needed etc.)
10	Any specific observation/ comments

F. Interaction with the investigator and co-investigator:

Key Informant(s)

	Department	Name	Designation	Brief observation (positive/neutral/negative)
1				
2				
3				

	**Multisite Active AEFI Sentinel Surveillance (MAASS) project:**<br><br>*As you are aware, the implementation of the MAASS network requires screening and identification of cases with suspected/confirmed diagnoses of a variety of clinical conditions, including AFP, ADEM, aseptic meningitis, sepsis, seizure, anaphylaxis, intussusception, UTI, malaria, Dengue and Kawasaki's disease.*
a	What strategies should be adopted to identify these patients?<br><br>*(How to search the cases and identify the suspected and confirmed cases?)*
b	Surveillance of varied clinical conditions mentioned above requires inter- and intra-departmental cooperation. How will you obtain support from the faculty/residents/nurses:<br><br>i) Other paediatric units<br><br>ii) Paediatric surgery department<br><br>iii) Neurology/Medicine departments<br><br>iv) Haematology department
c	For this project, a detailed history of vaccinations along with date of administration will be required for patients. How can this information be obtained?

d	In your view, what proportion of children <2 yrs of age admitted in the hospital have their vaccine cards available?	
	i) Available during the hospital stay	
	ii) Available at home (but not during hospitalization)	
	iii) Not available at all	
	iv) Feasibility of getting the immunization card, if not available at the time of hospitalization	
e	What is the risk of missing eligible cases during surveillance<br>*(Probe: emergency department /incomplete records/holidays)?*	
f	Do you anticipate any issues while obtaining consent from the parents/guardian and how may these challenges be addressed?<br>*(Consent shall be needed to collect information forms the parents regarding the socio-demography and other relevant information.)*	

G	What are the challenges anticipated by you while undertaking such a surveillance project?

G. Feedback about interaction with HOD, Paediatrics and/or Head of Institute

1	*Please brief the HoD and/or head of institute regarding the project objectives for MAASS and the type of support the investigator will require to accomplish tasks necessary for implementation of these projects. Please document key observations from your subsequent interaction with the HoD and/or Head of Institute:*<br><br>***a) Feedback from interaction with Head of Department:***<br><br>***b) Feedback from interaction with Head of Institute (Principal/ Dean/ Medical Superintendent)?***
