# Supplementary material for: Establishing a Multicenter Active Adverse Events Following Immunization Sentinel Surveillance Network Across 22 Tertiary Care Hospitals in India: Protocol for a Prospective Observational Study
Source: JMIR Res Protoc. 2025 Aug 8;14:e64050. doi: 10.2196/64050 (PMC12374136; doi:10.2196/64050)
Supplement: Multimedia Appendix 2 [file resprot_v14i1e64050_app2.pdf]

Appendix material A2: Outcome screening checklists

Screening Checklist-1. Any of the following:

1. Acute disseminated encephalomyelitis (ADEM)
2. Anaphylaxis
3. Aseptic Meningitis
4. Dengue
5. GBS
6. Intussusception
7. Kawasaki Disease
8. Malaria
9. Seizure
10. Sepsis
11. Thrombocytopenia
12. Urinary Tract Infection

Screening Checklist-2. Any two of the following:

1. Fever/Pyrexia
2. Crying excessively (for more than 6 hours) / Irritability
3. Headache/ Muscle pain/ Joint pain
4. Redness in eyes
5. Erythema/ Redness/ Dryness/ Fissure/ Peeling/ Cracking (of mouth/ oral cavity)
6. Redness/ Erythema/ Rashes (in arms or legs)
7. Skin peeling/ shedding/ desquamation of perianal or perineal region
8. Erythema, induration or crust formation at site of previous BCG vaccination
9. Swelling of palms and soles
10. Lymphadenopathy/ Lymph node enlargement
11. Dysuria (Pain/ crying during urination)
12. Pain in abdomen
13. Distension of abdomen/ Abdominal mass
14. Blood per rectum/ Rectal prolapse
15. Unconsciousness/ Loss of consciousness/ Altered sensorium/ Excessive sleepiness
16. Abdominal eye movement/ Weakness of eye movements/ Cranial nerve deficit/ Facial weakness
17. Loss of deterioration of speech after attaining (aphasia)
18. Bulging fontanelle
19. Nuchal/ neck rigidity

20. Ataxia (lack of voluntary coordination of movements/ frequent falling)
21. Sensory abnormality/ Decreased or absent reflexes/ Primitive reflexes present/ Any other neurologic finding

Screening Checklist-3. Any one of the following:

1. Purpura/ Ecchymosis/ Petechiae (red or purple spots on skin)/ Haemorrhage/ Bleeding/ Epistaxis
2. Low blood pressure requiring intravenous fluids, with or without vasopressors (dopamine, dobutamine, vasopressin, etc.)
3. Involuntary movements/ abnormal body movements/ Convulsions/ Generalized, tonic-clonic, atonic, motor manifestation.
4. Weakness of muscles strength/ Weakness of limbs/ Inability to walk
