# Supplementary material for: Establishing a Multicenter Active Adverse Events Following Immunization Sentinel Surveillance Network Across 22 Tertiary Care Hospitals in India: Protocol for a Prospective Observational Study
Source: JMIR Res Protoc. 2025 Aug 8;14:e64050. doi: 10.2196/64050 (PMC12374136; doi:10.2196/64050)
Supplement: Multimedia Appendix 3 [file resprot_v14i1e64050_app3.pdf]

Appendix material A3: Standardized case definitions for control conditions

Dengue

<u>Level 1 of diagnostic certainty:</u>

1) A case compatible dengue, with or without warning signs or severe dengue (defined in annexure to dengue definition below) **AND**

2) Laboratory confirmation of dengue infection using at least **one or more** of the following:

- Isolation of the dengue virus (Virus culture +VE) from serum, plasma, leucocytes. Demonstration of IgM antibody titre by ELISA positive in single serum sample at any time of the illness.
- Demonstration of dengue virus antigen in serum sample by NS1-ELISA from first day onwards at any time of the illness[1].
- IgG sero-conversion in paired sera after 2 weeks with four-fold increase of IgG titer.
- Detection of viral nucleic acid by polymerase chain reaction (PCR)

<u>Level 2 of diagnostic certainty:</u>

1) Level 2A of diagnostic certainty

A. A case compatible dengue, with or without warning signs or severe dengue (defined in annexure 1 below) AND

- **At least one** of the following: A positive RDT test **or** IgM positive **or** Serum IgG level by ELISA/ HI > 1280 after 5 days

2) Level 2B of diagnostic certainty

A. A case compatible dengue, with or without warning signs or severe dengue (defined in annexure 1 below) AND

B. Laboratory confirmation of dengue infection using criteria outlined in point number 2 of level 1 OR point number 2 of level 2A.

<u>Level 3 of diagnostic certainty</u>

1) A case of reported dengue **AND**

2) Occurrence of other confirmed cases of dengue fever in the locality **AND**

3) No other documented illness (e.g., malaria, sepsis, leukaemia) that can cause similar clinical manifestations as described in point 1 of level 1 diagnostic certainty criteria **AND**

4) Laboratory diagnostic test for Dengue not done.

---

[1] The NS1 should be done in the first 5 days of the illness and IgM should be done after 5 days of the illness.

Annexure Dengue: Clinical manifestations of Dengue [1]

1. ***Dengue without warning signs***: Acute febrile illness (perceived high fever or temperature >38.5 ^{0}C) of 2-7 days with **two or more** of the following:
    - Nausea, vomiting
    - Rash,
    - Aches and pains
    - positive tourniquet test[2],
    - leucopoenia (TWBC ≤5000 cells/mm3),
2. ***Dengue with warning signs***: Signs of dengue as described above, along with any of the following warning signs:
    - Abdominal pain or tenderness
    - Persistent vomiting
    - Clinical fluid accumulation
    - Mucosal bleed
    - Lethargy; restlessness
    - Liver enlargement >2cm
    - Increase in Haematocrit concurred with rapid decrease in platelet count
3. ***Severe dengue:*** Dengue with or without warning signs, in addition to the following signs

    i) Severe plasma leakage leading to:
    - Shock (DSS)
    - Fluid accumulation with respiratory distress

    ii) Severe bleeding as evaluated by clinician

    iii) Severe organ involvement
    - Liver: AST or ALT>=1000
    - CNS: Impaired consciousness
    - Heart and other organs

---

[2] The tourniquet test is performed by inflating a blood pressure cuff to a point midway between the systolic and diastolic pressures for five minutes. The test is considered positive when 10 or more petechiae per sq. inch are observed. In DHF the test usually gives a definite positive result with 20 petechiae or more. The test may be negative or only mildly positive in obese patients and during the phase of profound shock. It usually becomes positive, sometimes strongly positive after recovery from shock

Malaria

Level 1 of diagnostic certainty:

1. Any patient presenting with fever (more than 38.5 degrees) or a clinical history of fever in the preceding 48 hours / clinical suspicion of malaria

AND

2. Detection of malaria parasite by **any one** of the following two methods:
   - By light microscopy of thick and thin stained blood smears using Giemsa, Wright's or Field's strain using peripheral blood, within **24 hours** of suspected malaria [2, 3].

     OR
   - Detection of parasite using Polymerase Chain Reaction (PCR) based tests

Level 2 of diagnostic certainty:

1. Any patient presenting with fever (more than 38.5 degrees) or a clinical history of fever in the preceding 48 hours / clinical suspicion of malaria

AND

2. Tested positive for malaria parasite by using any Rapid Diagnostic Tests (RDTs).

Level 3 of diagnostic certainty:

1. Any patient presenting with fever (more than 38.5 degrees) or a clinical history of fever in the preceding 48 hours / clinical suspicion of malaria

AND

2. Residing in a malaria endemic area **OR** recently visited a malaria endemic area

AND

3. Tested negative for RDT and smear diagnostic test

AND

4. Investigations for other infection/ cause of fever negative

Note: *Other symptoms may include, but are not limited to chills and rigors, myalgia, arthralgia, anorexia, nausea, vomiting, running nose, other signs of respiratory infection, burning micturition and/or lower abdominal pain, skin rash/infections, abscess, painful swelling of joints, ear discharge, lymphadenopathy etc.[4]. Severe malaria is characterized by one or more of these additional features: impaired consciousness, repeated generalized convulsions, renal failure (serum creatinine >3 mg/dl), jaundice (serum bilirubin >3mg/dl), severe anaemia (Hb <5 g/dl), pulmonary oedema/acute respiratory distress syndrome, hypoglycaemia (plasma glucose <40 mg/dl), metabolic acidosis, circulatory collapse/shock (systolic BO<80 mm Hg, <50 mm Hg in children), abnormal bleeding and disseminated intravascular coagulation (DIC), haemoglobinuria, hyperpyrexia (temperature >106°C) etc.*

Sepsis

Level 1 of diagnostic certainty:

1) A case presenting with clinical manifestations of sepsis or illnesses in the sepsis continuum *(refer annexure to sepsis definition below)*, any one of the following [5, 6] : Sepsis or Severe Sepsis or Septic Shock

AND

2) Isolation/detection of pathogen(s) from sterile body fluids (except urine) using standardized techniques[7]:

- By culture OR
- By special stain in sample/ tissue OR
- By molecular technique(s) (RT PCR) OR
- Isolation of diphtheria from Pharynx sample

Level 2 of diagnostic certainty:

1) A case presenting with clinical manifestations of sepsis or illnesses in the sepsis continuum *(refer annexure to sepsis definition below)*, any one of the following [5, 6] : Sepsis or Severe Sepsis or Septic Shock

AND

2) Positive result for any of the following biomarkers/ serology tests[8]:

- Increase in the levels of C-reactive Protein > 10 mg/L in samples collected at 24 hours' intervals (quantitative method)
- Pro-calcitonin level higher than 2 ng/ml
- Serological test suggestive (quantitative by ELISA or immuno-turbidometry method) of the specific infections (i.e. salmonella, streptococcal infection, infectious mononucleosis, scrub typhus, etc.)
- Isolation of viral organism/antigen from other body fluid/sample (nasopharyngeal aspirate) using culture or direct isolation or PCR

Level 3 of diagnostic certainty:

- A case presenting with clinical manifestations of sepsis or illnesses in the sepsis continuum, any one of the following [5, 6] : Sepsis or Severe Sepsis or Septic Shock

Annexure Sepsis: Features of sepsis and illnesses in the sepsis continuum [5, 6]

1. **Sepsis:**

Features of **Systematic Inflammatory Response Syndrome (SIRS):** The presence of ***at least two*** of the following four criteria, *one of which must be abnormal temperature or leukocyte count*:

Core [oral or rectal] temperature of >38.5 °C or <36 °C **OR**

Axillary temperature >37.5°C or <36 °C

1.1. Leukocyte count elevated (>12000) or depressed (<4000) for age [not secondary to chemotherapy-induced leukopenia] or >10% immature neutrophils **AND**

1.2. Tachycardia, defined as heart rate >2 SD above normal for age(refer table below), in the absence of external stimulus, chronic drugs, or painful stimuli; or otherwise unexplained persistent elevation over a 0.5 h time-period or for children <1-year-old: bradycardia, defined as heart rate <10th percentile for age in absence of external vagal stimulus, β-blocker drugs, or congenital heart disease; or otherwise unexplained HR depression over a 0.5-h time-period **OR**

1.3. Tachypnoea > 2 SD above normal age or mechanical ventilation for an acute process not related to anaesthesia or any underlying neuromuscular disease **AND**

1.4. Presence of suspected or proven infection.

Category	Heart rate (beats/min) Mean (range)	Respiratory rate (breath/min)	Systolic BP, mmHg (range)	MAP-CVP (mmHg)
Up to 1 month	140 [100-190]	>60	<60	55
2 months to 1 year	130 [80-180]	>50	<70	60
1-5 years	80 [60-140]	>40	<70 + [2 x age in years)	65
6-10 years	80 [60-130]	>30	<70 + [2 x age in years)	65
>10 years	75 [60-100]	>30	<90	65

Age specific upper and/ or lower limits of heart rate to define tachycardia and bradycardia, respiratory rate to define tachypnoea, and systolic blood pressure to define hypotension.

2. **Infection:** A suspected or proven infection caused by any pathogen, or a clinical syndrome associated with a high probability of infection.

2.1. Evidence of infection on clinical examination, imaging, laboratory investigation

AND

2.2. Presence of petechial or purpuric rash, or purpura fulminans

OR

2.3. Imaging findings consistent with infection localized to any organ/site or perforation of any viscus

OR

2.4. Leukocytes in a normally sterile body fluid (blood, CSF, pleural fluid, ascitic fluid, pericardial fluid)

OR

2.5. Microbiology test positive from any sterile body fluid (blood, CSF, pleural fluid, ascitic fluid, pericardial fluid):

2.5.1. Positive culture OR

2.5.2. Positive tissue stain, OR

2.5.3. Positive organism using specific stain (gram stain/other) OR

2.5.4. Positive polymerase chain reaction test OR

2.5.5. Positive other molecular diagnostic techniques OR

2.5.6. Serology test for infections suggestive of rising titre (WIDAL for salmonella infection)

3. **Severe Sepsis:**

3.1. Features Sepsis AND

3.2. One of the following:

3.2.1. Cardiovascular organ dysfunction (specify as per the new guideline equal to septic shock) OR

3.2.2. Acute respiratory distress syndrome (list the details) OR

3.2.3. Two or more other organ dysfunction (define the organ dysfunction- Renal, Haematology, CNS, Liver)

4. **Septic Shock:**

4.1. Features of sepsis

AND

4.2. Presence of

4.2.1. Hypotension (Refer to table 1 above) **OR**

4.2.2. Need for vasoactive drug to maintain BP above fifth centile range [dopamine >5 mcg/kg/min or dobutamine, epinephrine, or norepinephrine at any dose]

OR

- Signs of hypoperfusion: ***Any three*** of the following
  - a) decreased pulse volume [weak or absent dorsalis pedis pulse],
  - b) capillary refilling time >3 s,
  - c) tachycardia heart rate as defined in core [rectal/oral] to peripheral [Skin-toe] temperature gap >3 °C,
  - d) urine output <1 ml/kg/h [<20 ml/h in >20 kg child]

e) altered sensorium

f) flash capillary refill/ bounding pulses

OR

4.3. Sepsis and cardiovascular organ dysfunction as defined below:

4.3.1. *Cardiovascular Dysfunction*

- Hypotension [systolic BP <70 mmHg in infant; <70 +2 × age after 1 year of age] **OR**
- Need for vasoactive drug to maintain BP above fifth centile range [dopamine >5 mcg/kg/min or dobutamine, epinephrine, or norepinephrine at any dose] **OR**
- Signs of hypoperfusion–any three of the following: decreased pulse volume [weak or absent dorsalis pedis pulse], capillary refilling time >3 s, flash capillary refill, wide pulse pressure, tachycardia [as defined above], core [rectal/oral] to peripheral [Skin-toe] temperature gap >3°C, and urine output <1 mL/kg/h [<20 mL/h in >20 kg child]. In early stage, there is an increase in heart rate and poor peripheral perfusion in form of weak pulse and prolonged capillary refill time. Hypotension occurs late and may lead to precipitous cardiac arrest.

4.3.2. Respiratory Dysfunction

- Proven need for supplemental oxygen(c) or >50% FIO2 to maintain saturation >92% **OR**
- Need for non-elective mechanical ventilation **OR**
- PaO2/FIO2 <300 in absence of cyanotic heart disease or pre-existing lung disease **OR**
- PaCO2 >65 torr or 20 mmHg over baseline PaCO2

4.3.3. Neurologic Dysfunction

- Glasgow Coma Score <11 **OR**
- Decreased or altered mental status with a decrease in Glasgow Coma Score >3 points from abnormal baseline

4.3.4. Hematologic Dysfunction

- Platelet count <80000/mm3 or a decline of 50% in platelet count from highest value recorded over the past 3 days [for chronic haematology/oncology patients] **OR**
- International normalized ratio >2

4.3.5. Renal Dysfunction

- Serum creatinine >1 mg/dL

4.3.6. Hepatic Dysfunction

- Total bilirubin >4 mg/dL or, alanine transaminase 2 × upper limit of normal for age

Urinary Tract Infection

Level 1 of diagnostic certainty:

1. A case presenting with fever (more than 38.5°C), without an identifiable source of infection **OR** symptoms referable to urinary tract in absence of fever [9, 10]

   AND

2. Demonstration of pyuria defined as:
   - 10 leucocytes per mm^3 in fresh, un-centrifuged sample [11, 12] **OR**
   - >5 leucocytes per high power field in a centrifuged [13] sample or approximately 25 WBCs/µl [9]

   AND

3. Demonstration of significant bacteriuria by urine culture, defined as [9, 13]
   - colony count of $>10^5$ CFU/ml of a single species uropathogen (see list in annexure to UTI below) in a fresh, un-centrifuged, mid-stream clean catch sample **OR**
   - colony count of $>5x10^4$ CFU/ml of a single species uropathogen in a fresh, un-centrifuged, sample collected by urethral catheterization **OR**
   - Any number of single species uropathogens isolated in a fresh, un-centrifuged, sample collected by suprapubic aspiration

Level 2 of diagnostic certainty:

A case presenting with fever (more than 38.5°C), without an identifiable source of infection OR symptoms referable to urinary tract in absence of fever [9, 10] **AND any one of the following:**

2 A) Demonstration of significant bacteriuria by urine culture as defined below:

- colony count of $>10^5$ CFU/ml of a single species uropathogens in a fresh, un-centrifuged, mid-stream clean catch sample **OR**
- colony count of $>5x10^4$ CFU/ml of a single species uropathogens in a fresh, un-centrifuged, sample collected by urethral catheterization **OR**
- Any number of single species uropathogens isolated in a fresh, un-centrifuged, sample collected by suprapubic aspiration

OR

2 B) Demonstration of pyuria **and** bacteriuria as follows:

i) Demonstration of pyuria defined by **any one** of the following:

- 10 leucocytes per mm^3 in fresh, un-centrifuged sample **OR**
- >5 leucocytes per high power field in a centrifuged sample or approximately 25 WBCs/µl **OR**
- Positive Leucocyte esterase test

AND

ii) Demonstration of bacteriuria defined by **any one** of the following:

- Urine culture: single species uropathogen with a colony count of >10^3 in a fresh, un-centrifuged, sample collected by urethral catheterization or a colony count of > 10^4 CFU/ml in a fresh, un-centrifuged mid-stream clean catch sample **OR**
- Positive urine microscopy for bacteria **OR**
- Positive Nitrite test

Level 3 of diagnostic certainty

1) A case presenting with fever, without an identifiable source of infection **OR** symptoms referable to urinary tract in absence of fever [9, 10]

AND

2) In the presence of sterile urine culture due to prior antibiotic use positive results for **any one** of the following tests in urine:

a) Demonstration of pyuria defined by any one of the following:

- 10 leucocytes per mm^3 in fresh, un-centrifuged sample
- >5 leucocytes per high power field in a centrifuged sample or approximately 25 WBCs/µl
- Positive Leucocyte esterase test

OR

b) Demonstration of bacteriuria by **any one** of the following:

- Positive urine microscopy for bacteria
- Positive Nitrite test

Annexure UTI: Common uropathogens as identified by the online textbook of urology include [14]

**Pathogen**	%
Gram negative pathogens	
E. Coli	77%
Proteus mirabilis	5%
Klebsiella pneumoniae	2-3%
Enterobacter spp	1%
Citrobacter spp	1%
Other Enterobacteriaceae	
Gram positive bacteria	
Staphylococcus saprophyticus	3%
Staphylococcus aureus	2%
Other staphylococci	4%
Enterococcus spp	3%
Streptococcus spp	1%

Other pathogens include, gram-negative anaerobic bacteria such as peptococcus and peptostreptococcus, Neisseria gonorrhoea, Mycobacterium tuberculosis, fungi (most commonly Candida species, followed by Aspergillus) and parasites such as Schistosoma haematobium.

References

1. WHO, *Dengue guidelines for diagnosis, treatment, prevention and control : new edition*. 2009, World Health Organization: Geneva.
2. Kundu, R., et al., *Diagnosis and management of malaria in children: recommendations and IAP plan of action.* Indian Pediatr, 2005. **42**(11): p. 1101-14.
3. Bell, D., C. Wongsrichanalai, and J.W. Barnwell, *Ensuring quality and access for malaria diagnosis: how can it be achieved?* Nat Rev Microbiol, 2006. **4**(9 Suppl): p. S7-20.
4. WHO, *WHO malaria terminology*. 2016, World Health Organization: Geneva.
5. Goldstein, B., B. Giroir, and A. Randolph, *International pediatric sepsis consensus conference: definitions for sepsis and organ dysfunction in pediatrics.* Pediatr Crit Care Med, 2005. **6**(1): p. 2-8.
6. Khilnani, P., et al., *Pediatric Sepsis Guidelines: Summary for resource-limited countries.* Indian journal of critical care medicine : peer-reviewed, official publication of Indian Society of Critical Care Medicine, 2010. **14**(1): p. 41-52.
7. Kirn, T.J. and M.P. Weinstein, *Update on blood cultures: how to obtain, process, report, and interpret.* Clin Microbiol Infect, 2013. **19**(6): p. 513-20.
8. Davis, A.L., et al., *American College of Critical Care Medicine Clinical Practice Parameters for Hemodynamic Support of Pediatric and Neonatal Septic Shock.* Crit Care Med, 2017. **45**(6): p. 1061-1093.
9. Roberts, K.B., *Urinary tract infection: clinical practice guideline for the diagnosis and management of the initial UTI in febrile infants and children 2 to 24 months.* Pediatrics, 2011. **128**(3): p. 595-610.
10. Becknell, B., et al., *The diagnosis, evaluation and treatment of acute and recurrent pediatric urinary tract infections.* Expert Rev Anti Infect Ther, 2015. **13**(1): p. 81-90.
11. Delanghe, J. and M. Speeckaert, *Preanalytical requirements of urinalysis.* Biochemia medica, 2014. **24**(1): p. 89-104.
12. Doern, C.D. and S.E. Richardson, *Diagnosis of Urinary Tract Infections in Children.* Journal of clinical microbiology, 2016. **54**(9): p. 2233-2242.
13. Vijayakumar, M., et al., *Revised statement on management of urinary tract infections.* Indian Pediatr, 2011. **48**(9): p. 709-17.
14. Flores-Mireles, A.L., et al., *Urinary tract infections: epidemiology, mechanisms of infection and treatment options.* Nature reviews. Microbiology, 2015. **13**(5): p. 269-284.
